# Paeonol at Certain Doses Alleviates Aggressive and Anxiety-Like Behaviours in Two Premenstrual Dysphoric Disorder Rat Models

**DOI:** 10.3389/fpsyt.2020.00295

**Published:** 2020-04-15

**Authors:** Hao Zhang, Xiwen Geng, Zifa Li, Yaqiong Li, Kaiyong Xu, Hongyun Wu, Jinlu Xie, Peng Sun, Sheng Wei, Mingqi Qiao

**Affiliations:** ^1^ Key Laboratory of Traditional Chinese Medicine Classical Theory, Ministry of Education, Shandong University of Traditional Chinese Medicine, Ji’nan, China; ^2^ Experimental Center, Shandong University of Traditional Chinese Medicine, Ji’nan, China; ^3^ Bozhou Institute of Traditional Chinese Medicine, Anhui Academy of Chinese Medicine Sciences, Bozhou, China; ^4^ No. 3 Department of Encephalopathy, Affiliated Hospital of Shandong University of Traditional Chinese Medicine, Ji’nan, China; ^5^ Key Laboratory of Vector Biology and Pathogen Control of Zhejiang, School of Medicine, Huzhou University, Huzhou Central Hospital, Huzhou, China

**Keywords:** paeonol, premenstrual dysphoric disorder, rat models, resident intruder, progesterone withdrawal

## Abstract

Premenstrual dysphoric disorder (PMDD) is a severe form of premenstrual syndrome (PMS), a common mental health disturbance associated with several periodic psychological symptoms in women. Selective serotonin reuptake inhibitors (SSRIs) are the first-line treatment for PMS/PMDD patients; however, side effects are inevitable, especially in long-term treatment. In previous studies, the natural compound paeonol in Moutan Cortex was found to play effective roles in central nervous system disorders with its anti-inflammatory, anti-oxidant, and neuroprotective effects. Consequently, we assume that paeonol might produce positive effects in the treatment of PMS/PMDD. In this study, the open-field test (OFT) and elevated plus maze (EPM) and light dark box (LDB) tests were performed in mice to determine the optimal dose of paeonol for treating anxiety. Then, paeonol was used to treat the progesterone withdrawal (PWD) and resident intruder paradigm (RIP) rat models of PMDD. Using these two reliable models, the OFT and EPM, LDB, and composite aggressive tests were performed to evaluate the effect of the drug on behavioural symptoms of PMDD. From the dosage screening results, the optimal anti-anxiety dose of paeonol was identified as 17.5 mg/kg/d for 7 days. With regard to the effect of paeonol on PMDD rat models, a significantly improvement was found in the behavioural symptoms, but the effective dose varied in different models. For the PWD model rats, treatment with 6.05 mg/kg paeonol could significantly improve anxiety and irritability, while that with 24.23 mg/kg paeonol resulted in anxiety-like effects in behavioural tests. In RIP model rats, treatment with 12.11 mg/kg paeonol demonstrated excellent effects in improving anxiety, particularly irritable emotional behaviour. In conclusion, our study indicates that paeonol is a potential therapeutic compound for PMS/PMDD; it is a drug option that helps establish dosage guidance for treatment of this condition.

## Introduction

Women experiencing premenstrual syndrome (PMS), one of the most common mental health disturbances, have several characteristic psychological symptoms including anger, irritability, depressed mood, and anxiety (1). A severe form of PMS, afflicting up to 5–8% of patients, was termed as premenstrual dysphoric disorder (PMDD) in the fifth edition of the Diagnostic and Statistical Manual of Mental Disorders. In women with PMDD, symptoms occur during the luteal phase of each menstrual cycle and disappear by the end of menstruation.

In clinical trials, the first-line treatment for PMS/PMDD patients is antidepressants in the form of selective serotonin reuptake inhibitors (SSRIs), such as clomipramine, escitalopram, fluoxetine, sertraline, and paroxetine (2, 3). SSRIs have played a significant role in reducing both mood symptoms and somatic complaints; thus, they have been considered as the gold standard in the treatment of this disorder (4, 5). However, a re-evaluation published in a journal, *CNS Drugs*, in 2006 found that although SSRIs were more effective in PMDD than a placebo, the response rate was less than 60%, which was far from satisfactory (6). In addition, side effects like nausea and headache are common, particularly in long-term treatment (7). A recent report (8) indicated that short-term low dose fluoxetine could prevent anxiety-like behaviour related to the oestrous cycle in female rats; however, evaluation of its effect on depression and irritability is lacking. Therefore, there is an urgent need to develop new drug treatment methods to improve the therapeutic management of PMS/PMDD. Traditional Chinese medicine has shown remarkable efficacy in the treatment of PMDD (9). The natural compound paeonol is an active constituent of Moutan Cortex. It has been reported that paeonol has a neuroprotective effect by the inflammatory processes mediated by microglial activation (10, 11) and plays a role in central nervous system disorders including depression (12, 13). Based on this evidence, we assume that paeonol might produce positive effects in the treatment of PMS/PMDD.

As reported, long-term exposure to exogenous progesterone along with its abrupt withdrawal has been a reliable method for establishing a rodent PMDD model. The use of progesterone withdrawal (PWD) protocols sufficiently induces depression and a negative mood during the luteal phase (14–17). Besides, resident intruder paradigm (RIP) is a common method used to establish a PMDD rat model. By performing ovariectomy and providing exogenous hormone supplementation, the normal cyclical release of hormones was mimicked. The cycle-dependent aggressive behaviours (irritability and anger) were elicited by introducing an intruder rat into a resident rat cage, which has been confirmed in several reports (18–20).

In this study, the open-field test (OFT) and elevated plus maze (EPM) and light dark box (LDB) tests were initially performed in mice to determine the optimal dose of paeonol for treatment of anxiety. Then, paeonol was used to treat rats subjected to PWD and RIP stress. To evaluate the drug effect precisely using these two recognised and reliable PMDD models, a series of behavioural assessment tests was performed including the OFT and EPM, LDB, and composite aggressive tests.

## Materials and Methods

### Ethical Approval

All experiments were approved by the ethics review board of Shandong University of Traditional Chinese Medicine (No. DWSY201703013) and conducted in accordance with the National Institutes of Health Guidelines for the Care and Use of Laboratory Animals.

### Subjects

To determine the optimal dose of paeonol for the treatment of anxiety, C57BL/6J male mice (6–8 weeks old, weighing 18–22 g) were used in the OFT and EPM and LDB tests. Then, the PMDD-PWD model and the resident intruder model of PMDD were established to evaluate the drug effect on female Wistar rats (6–7 weeks old, weighing 140–160 g). All animals were purchased from the Vital River Laboratories (Beijing, China) and housed at 21 ± 1°C and 55% relative humidity under a 12:12 h light/dark cycle with food and water available *ad libitum*. The animals were habituated to maintenance conditions for 1 week and handled daily to eliminate the human factor.

### Experimental Design and Drug Treatment Regimen

In the dosage screening against anxiety, mice were randomly divided into seven groups: one control group (received saline with 0.5% sodium carboxymethyl cellulose [CMC-Na]), five paeonol groups (received 8.75, 17.5, 35, 70, and 140 mg/kg/d of paeonol, respectively), and one positive control group (received 2 mg/kg/d of diazepam). Paeonol (purity > 98%, from the pharmaceutical laboratory of Shandong University of Traditional Chinese Medicine) and diazepam (Jichuan Pharmaceutical Group Co., Ltd., H32021083) used for the positive control group were dissolved in saline-0.5% CMC-Na. All drugs were administered through gavage once daily at approximately 9:00 am for 7 consecutive days. The behavioural tests were conducted after the final administration. To make the results more accurate, each mouse was used for only one behavioural test, and the other studies were conducted in separate cohorts.

In order to determine the optimal dose of paeonol for the treatment of anxiety, the PMDD-PWD model and the resident intruder model of PMDD were used in the pharmacodynamic evaluation. The rats were divided into six groups: one control group (normal rats received saline), one PMDD group (PMDD rats received saline), three paeonol groups (each PMDD rat received low, moderate, and high doses of paeonol), and one positive control group (PMDD rat received 2.7 mg/kg/d of fluoxetine). As fluoxetine is a first-line regimen used in the clinical treatment for PMDD, which is different from the anti-anxiety drug, diazepam used in the dosage screening experiment as previously mentioned, we used fluoxetine in the positive control group to be consistent with the clinical treatment. Based on the optimal dose of paeonol against anxiety in mice, the dose used in PMDD rats was calculated as follows according to the third edition of the Pharmacologic Experimental Methodology (Editor-in-chief: Shuyun, People’s Medical Publishing House, Beijing 2002) and dissolved in saline with 0.5% CMC-Na: moderate dose=optimal dose in mice/9.1×6.3; low dose=moderate dose/2; high dose=moderate dose×2. Fluoxetine (Lilly Suzhou Pharmaceutical Co. Ltd., J20160029) was dissolved in saline-0.5% CMC-Na. All drugs were administered intragastrically once daily at approximately 9:00 am, for 16 consecutive days in the PMDD-PWD rat model and for 12 consecutive days in the resident intruder model of PMDD.

### PMDD-PWD Rat Model

**Figure 1:**
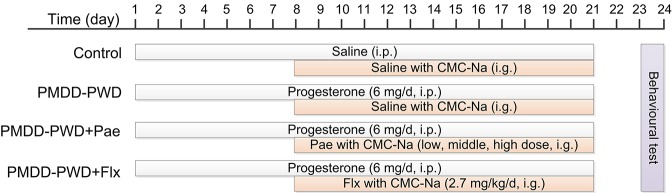
Schedule for the progesterone withdrawal protocol, drug administration, and behavioural testing. PMDD-PWD, premenstrual dysphoric disorder-progesterone withdrawal; i.p., intraperitoneal injection; i.g., intragastric administration; CMC-Na, sodium carboxymethyl cellulose; Pae, paeonol; Flx, fluoxetine.

To induce the PMDD-PWD model (**Figure 1**), the rats were administered intraperitoneal progesterone (Sigma, 101327062) injections (6 mg/d for each rat, dissolved in saline) for 21 consecutive days. Then, they were subjected to PWD as it could reliably increase depression-like behaviour according to a previous study (16). The drug treatment (fluoxetine or paeonol) was started from the 8^th^ day after the first progesterone administration to the day of the behavioural tests. The behavioural tests were conducted 48–72 h after the final progesterone administration. Each rat was used for only one behavioural test, and the other studies were conducted in separate cohorts.

### Resident Intruder Paradigm Model of PMDD (PMDD-RIP)

**Figure 2:**
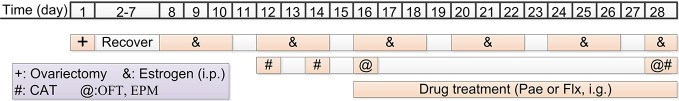
Schedule for the resident intruder model of premenstrual dysphoric disorder (PMDD) drug administration and behavioural testing. i.p., intraperitoneal injection; i.g., intragastric administration; Pae, paeonol; Flx, fluoxetine; CAT, composite aggressive test; OFT, open-field test; EPM, elevated plus maze.

A typical rat oestrus is divided into four phases: non-receptive phase (3 days, including metoestrus, dioestrus I, and dioestrus II) and receptive phase (1-day, pro-oestrus/oestrus). Ovulation occurs in the receptive phase. In order to bring the subjects into consistent oestrus, the ovary was removed and exogenous oestrogen was supplemented to induce the periodic oestrus (**Figure 2**). In the ovariectomy procedure, the rats were anaesthetised using an intraperitoneal injection of 2% pentobarbital sodium (60 mg/kg) and immobilised on the rat board under aseptic conditions. Then, bilateral ligation of uterine tubes and removal of ovaries were performed, and the incision was sutured. The subjects were allowed 7 days to recover from the surgery.

Seven days after the surgery, exogenous oestrogen was administered to induce the oestrous cycle according to the following regimen (18): at 11:30 am on the 8^th^ day, 0.5 ug oestradiol benzoate (McLean Shanghai trading Co. Ltd., 10042617; dissolved in 0.1 ml saline) was injected subcutaneously; 32 h later, 0.5 ug oestradiol (McLean Shanghai trading Co. Ltd., 10006920; dissolved in 0.1 ml saline) was injected subcutaneously; 44 h later, 0.5 mg progesterone (dissolved in 0.1 ml saline) was injected subcutaneously. The exogenous oestrogen injection regimen was repeated five times with a 1-day interval. The first, second, and third days of exogenous oestrogen injection corresponded to the dioestrus I, dioestrus II, and receptive phase, respectively.

During the second injection of exogenous oestrogen, a composite aggressive test was performed during the receptive and dioestrus I (18, 21) phase. The test was conducted at 12:30–15:30 pm under a dim (< 2 lux) lighting condition. After 15 min of habituation of the resident rat, an ovariectomised stimulus female intruder was introduced into the resident’s cage and this was video recorded for 10 min. During this period, we scored the aggressive behaviour (attacking, biting, and jumping at the intruder) (20). The composite aggressive score was defined as the attacking instances + 0.2×attacking time (s) + biting instances + 0.2×jumping time (s). Then, the animals were divided into different groups according to the value of the composite aggressive score in dioestrus I (12^th^ day) minus the score in the receptive phase (14^th^ day). The animals were sorted by their scores from high to low, and the first 30% were divided into the PMDD model groups (the PMDD group and paeonol groups) and the last 30% were divided into the control group. The rest of the animals were eliminated (20).

### Open-Field Test

The animals were placed in a square apparatus (50 cm × 50 cm for mice and 100 cm × 100 cm for rats), with the arena divided into nine equal squares for 6 min. Each mouse or rat was placed individually into the centre and permitted free exploration. With the XR-Super Maze tracking system (Shanghai Xinsoft Information Technology Co. Ltd.), the total distance, central area distance, time at central area, and the instances of standing on the hind legs (rearings) were recorded during the test time (22). The arena was cleaned with 70% ethanol after every trial. Anxiety-like behaviour was defined when the animal spent more time near the edges of the box than at the centre.

### Elevated Plus Maze

The EPM test was conducted using the XR-Super Maze tracking system and a polypropylene plastic cruciform apparatus that was elevated 76 cm above the floor. The cruciform box consisted of two open arms, two closed arms (30 cm × 5 cm for mice and 50 cm × 10 cm for rats), and a central platform (the junction area, 5 cm × 5 cm for mice and 10 cm × 10 cm for rats). Each animal was placed on the central platform with the head towards the open arm, and the behaviour was recorded within 5 min, including the open-arm entry times (OE), closed arm entry times (CE), time in the open arm (OT, s), time in the closed arm (CT, s), total distance, and distance in the closed arm. Then, the OE% and OT% were calculated as follows: OE%=OE/(OE+CE)×100%, OT%=OT/(OT+CT)×100%. Higher anxiety was indicated by a lower frequency of entry into the open arms and lesser time of stay (23). The arena was cleaned with 70% ethanol after every trial.

### Light Dark Box

The light dark box consisted of two chambers of the same size (25 cm × 25 cm × 30 cm), with the dark and light compartments separated by a door (6.5 cm × 6.5 cm). The animals were placed in the centre of the light box and allowed to move freely between the two chambers within 5 min. A video tracking system (XR-Super Maze) was used to record the total distance, time spent in the light box, light box entry times, and the distance in the light box (24). As the rodents have an innate aversion to light areas and were allowed spontaneous exploration, the indexes previously mentioned could indicate their anxiety-like behaviour.

### Statistical Analysis

Statistical analysis was performed using Graph Pad Prism 7.0.4 software (GraphPad Software, Inc., San Diego, CA, USA). First, the Kolmogorov-Smirnov and the Levene tests were performed to test for normal distribution and homogeneity of variance. One-tailed unpaired *t*-tests were performed for two-group comparison, while one-way analysis of variance followed by Bonferroni’s test was used to compare the differences among three or more groups. Data were presented as mean ± standard error of the mean. For all analyses, P values <0.05 were considered signiﬁcant, and the level of signiﬁcance was described as *p < 0.05, **p < 0.01, ***p < 0.001 and ****p < 0.0001.

## Results

### Dosage Screening of Paeonol Against Anxiety

**Figure 3:**
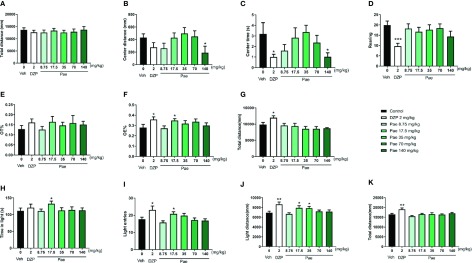
Results of the dosage screening of paeonol against anxiety. **(A)** Total distance in the open-field test (OFT). **(B)** Central area distance in the OFT. **(C)** Time in the central area in the OFT. **(D)** Instances of standing on the hind legs (rearings) in the OFT. **(E)** Percentage of time in the open arm in the elevated plus maze (EPM) test. **(F)** Percentage of open-arm entry times in the EPM test. **(G)** Total distance in the EPM test. **(H)** Time spent in the light compartment in the light dark box (LDB) test. **(I)** Light compartment entry times in the LDB test. **(J)** Distance in the light compartment in the LDB test. **(K)** Total distance in the LDB test. Veh, vehicle; Dzp, diazepam; Pae, paeonol; OFT, open-field test; EPM, elevated plus maze; LDB, light dark box; *p < 0.05, **p < 0.01, ***p < 0.001 compared with the control group. Nine animals were used in each group.

To determine the appropriate paeonol dosage for the pharmacodynamic evaluation of PMDD animals, we first designed the experiment using mice treated with different dosages of paeonol. Specifically, in the OFT, as shown in **Figures 3A–D**, diazepam intervention decreased the time in the central area (p=0.0319) and rearing times (p=0.0004) compared with the control group, and 140 mg/kg/d of paeonol decreased the central area distance (p=0.0298) and time in the central area (p=0.0365). Then, in the EPM test (**Figures 3E–G**), the OE% of mice was increased by diazepam (p=0.0348) and 17.5 mg/kg/d paeonol (p=0.0363); diazepam also increased the total distance (p=0.0180). Lastly, in the LDB test (**Figures 3H–K**), diazepam increased the light entries (p=0.0248), light distance (p=0.0048), and total distance (p=0.0064), which confirmed its anti-anxiety effect; 17.5 mg/kg/d paeonol also increased the time in the light box (p=0.0450), light entries (p=0.0442), and light distance (p=0.0483). Furthermore, 35 mg/kg/d paeonol could increase the light distance (p=0.0376). Using the three classical anti-anxiety behavioural tests including the OFT, EPM, and LDB, the effects of the different paeonol dosages were evaluated, and the optimal anti-anxiety dosage was confirmed as 17.5 mg/kg/d.

### Behavioural Identification of the PMDD-PWD Rat Model

**Figure 4:**
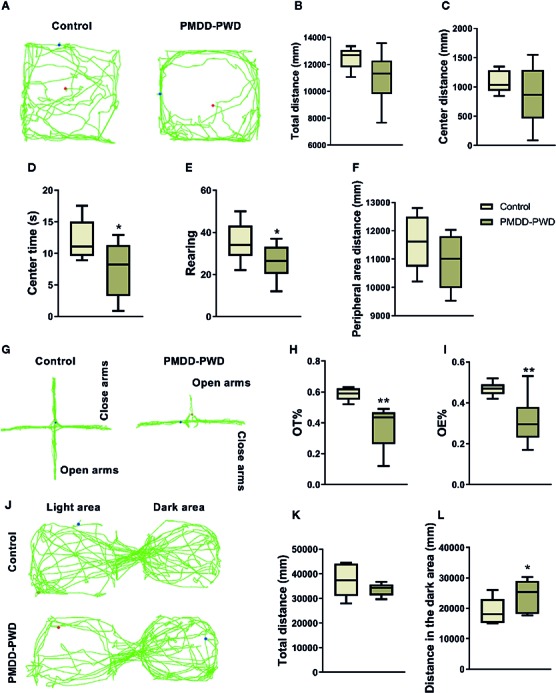
Results of the behavioural identification in the premenstrual dysphoric disorder-progesterone withdrawal (PMDD-PWD) rat model. **(A)** The representative trajectory diagram of control and PMDD-PWD rats in the open-field test (OFT). **(B)** Total distance in the OFT. **(C)** Central area distance in the OFT. **(D)** Time in the central area in the OFT. **(E)** Instances of standing on the hind legs (rearings) in the OFT. **(F)** Peripheral area distance in the OFT. **(G)** The representative trajectory diagram of control and PMDD-PWD rats in the elevated plus maze (EPM) test. **(H)** Percentage of time in the open arm (OT%) in the EPM test. **(I)** Percentage of open-arm entry times (OE) in the EPM test. **(J)** The representative trajectory diagram of control and PMDD-PWD rats in the light dark box (LDB) test. **(K)** Total distance in the LDB test. **(L)** Distance in the dark compartment in the LDB test. *p < 0.05, **p < 0.01 compared with the control group. PMDD-PWD, premenstrual dysphoric disorder-progesterone withdrawal; OFT, open-field test; EPM, elevated plus maze; LDB, light dark box; OE, open-arm entry times; OT, time in the open arm.

In a manner similar to that of the dosage screening experiment previously mentioned, behavioural tests including the OFT, EPM, and LDB were also used in the behavioural identification of the PMDD-PWD rat model. In **Figure 4**, compared with control rats (n=12), PMDD-PWD rats showed decreased time in the central area (n=12, p=0.0326) and rearing number (p=0.0478) in the OFT (**Figures 4A–F**). In the EPM test, PMDD-PWD rats tended to stay in the closed arms, thereby decreasing the OT% (p=0.0054) and OE% (p=0.0061) (**Figures 4G–I**). In addition, PMDD-PWD rats also showed significantly increased distance in the dark area (p=0.0485) in the LDB test (**Figures 4J–L**). These results showed that the PMDD-PWD model was established successfully because the animals significantly demonstrated anxiety-like behaviour.

### Behavioural Identification of the Resident Intruder Model of PMDD

**Figure 5:**
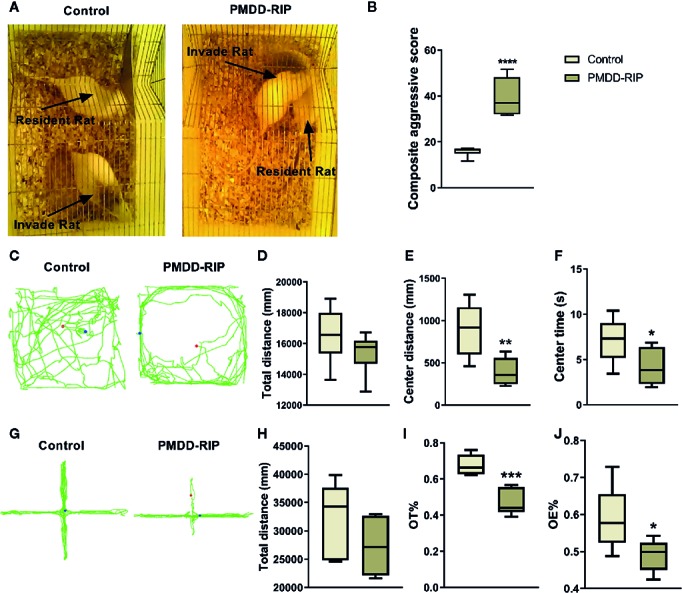
Results of the behavioural identification in the resident intruder model of premenstrual dysphoric disorder (PMDD). **(A)** The representative photograph of control and PMDD-RIP rats in the composite aggressive test. **(B)** Composite aggressive score in the dioestrus I phase. **(C)** The representative trajectory diagram of control and PMDD-RIP rats in the OFT. **(D)** Total distance in the OFT. **(E)** Central area distance in the OFT. **(F)** Time in the central area in the OFT. **(G)** The representative trajectory diagram of control and PMDD-RIP rats in the EPM test. **(H)** Total distance in the EPM test. **(I)** Percentage of time in the open arm (OT%) in the EPM test. **(J)** Percentage of open-arm entry times (OE) in the EPM test. *p < 0.05, **p < 0.01, ***p < 0.001, ****p < 0.0001 compared with the control group. PMDD-RIP, premenstrual dysphoric disorder-resident intruder paradigm; OFT, open-field test; EPM, elevated plus maze; OE, open-arm entry times; OT, time in the open arm.

The composite aggressive test, OFT, and EPM test were designed to evaluate the behavioural phenotypes of PMDD-RIP animals. In the composite aggressive test (**Figures 5A, B**), the PMDD-RIP rats (n=12) showed a significantly higher composite aggressive score than that of the control rats (n=12, p < 0.0001). PMDD-RIP rats also showed a shorter distance in the central area (p=0.0058) and reduced time (p=0.0465) in the central area in the OFT (**Figures 5C–F**); however, the total distance remained unchanged. In the EPM test (**Figures 5G–J**), the OT% (p=0.0003) and OE% (p=0.0301) were all decreased in PMDD-RIP rats. All these results indicated that we established a successful and reliable resident intruder model of PMDD because of the significant anxiety-like behaviour.

### Paeonol Improved the PMDD-Like Behaviour in Rat Models

#### The Effect of Paeonol on the PMDD-PWD Rat Model

**Figure 6:**
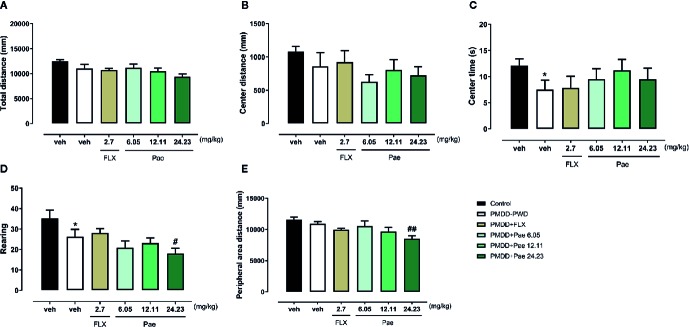
Results of the effect of paeonol on the premenstrual dysphoric disorder-progesterone withdrawal (PMDD-PWD) rat model in the OFT. **(A)** Total distance in the OFT. **(B)** Central area distance in the OFT. **(C)** Time in the central area in the OFT. **(D)** Instances of standing on the hind legs (rearings) in the OFT. **(E)** Peripheral area distance in the OFT. PMDD-PWD, premenstrual dysphoric disorder-progesterone withdrawal; OFT, open-field test; Flx, fluoxetine; Pae, paeonol; *p < 0.05 compared with the control group; ^#^p < 0.05 compared with the PMDD-PWD group; ^##^p < 0.01 compared with the PMDD-PWD group. Twelve animals were used in each group.

According to the dosage screening results of paeonol against anxiety, the dose of paeonol in rats was determined as follows: moderate dose=12.11 mg/kg/d, low dose=6.05 mg/kg/d, and high dose=24.23 mg/kg/d. In the OFT results as shown in **Figure 6**, the drug effect was not very obvious, except that 24.23 mg/kg paeonol decreased the rearing number (p=0.0478) and the peripheral area distance (p=0.0017) compared with PMDD-PWD rats.

**Figure 7:**
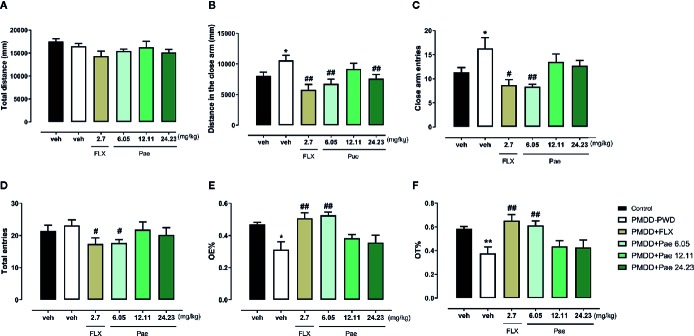
Results of the effect of paeonol on the premenstrual dysphoric disorder-progesterone withdrawal (PMDD-PWD) rat model in the elevated plus maze (EPM) test. **(A)** Total distance in the EPM test. **(B)** Distance in the closed arm in the EPM test. **(C)** Closed arm entry times in the EPM test. **(D)** Total entry times in the EPM test. **(E)** Percentage of time in the open arm in the EPM test. **(F)** Percentage of open-arm entry times in the EPM test. PMDD-PWD, premenstrual dysphoric disorder-progesterone withdrawal; EPM, elevated plus maze; Flx, fluoxetine; Pae, paeonol; *p < 0.05 compared with the control group; **p < 0.01 compared with the control group; ^#^p < 0.05 compared with the PMDD-PWD group; ^##^p < 0.01 compared with the PMDD-PWD group. Twelve animals were used in each group.

In the EPM test (**Figure 7**), 6.05 mg/kg paeonol showed significant anti-anxiety effects: the distance in the closed arm (p=0.0076), CE (p=0.0059), total entry times (p=0.0205), OE% (p=0.0024), and OT% (p=0.0059) were all reversed to normal level compared with PMDD-PWD rats receiving fluoxetine treatment (p=0.0026, 0.0121, 0.0484, 0.0091, and 0.0048, respectively). Likewise, 24.23 mg/kg paeonol also reversed the distance in the closed arm (p=0.0097).

**Figure 8:**
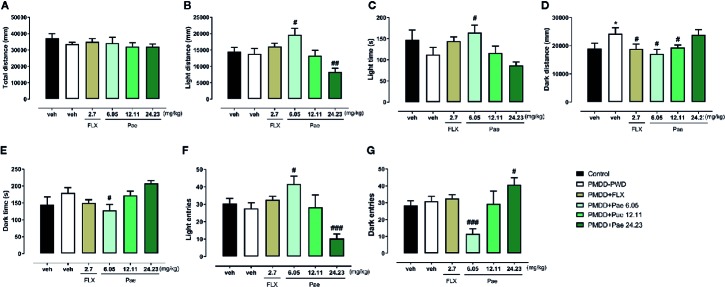
Results of the effect of paeonol on the premenstrual dysphoric disorder-progesterone withdrawal (PMDD-PWD) rat model in the light dark box (LDB) test. **(A)** Total distance in the LDB test. **(B)** Distance in the light box in the LDB test. **(C)** Time in the light box in the LDB test. **(D)** Distance in the dark box in the LDB test. **(E)** Time in the dark box in the LDB test. **(F)** Entry times to the light box in the LDB test. **(G)** Entry times to the dark box in the LDB test. PMDD-PWD, premenstrual dysphoric disorder-progesterone withdrawal; LDB, light dark box; Flx, fluoxetine; Pae, paeonol; *p < 0.05 compared with control group; ^#^p < 0.05 compared with the PMDD-PWD group; ^##^p < 0.01 compared with the PMDD-PWD group; ^###^p < 0.001 compared with the PMDD-PWD group. Twelve animals were used in each group.

In the LDB test shown in **Figure 8**, the total distance was not affected by the PMDD model or drug treatment. However, with respect to the light distance, 6.05 mg/kg paeonol increased the light distance (p=0.0223) compared with the PMDD-PWD group, while 24.23 mg/kg paeonol decreased this distance (p=0.0078); 6.05 mg/kg paeonol also increased the light time (p=0.0298) and decreased the dark time (p=0.0283). With respect to the dark distance, the fluoxetine group (p=0.0373), 6.05 mg/kg paeonol group (p=0.0109), and 12.11 mg/kg paeonol group (p=0.0225) yielded lower values compared with the PMDD-PWD group. The light entry times were increased with 6.05 mg/kg paeonol (p=0.0146) and decreased with 24.23 mg/kg paeonol (p=0.0009). Conversely, the dark entry times were decreased with 6.05 mg/kg paeonol treatment (p=0.0005) and increased with 24.23 mg/kg paeonol (p=0.0440).

#### The Effect of Paeonol on the Resident Intruder Model of PMDD

**Figure 9:**
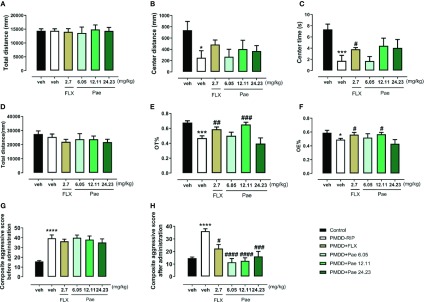
Results of the effect of paeonol on the resident intruder model of premenstrual dysphoric disorder (PMDD) in the open-field test (OFT) and elevated plus maze (EPM) and composite aggressive tests. **(A)** Total distance in the OFT. **(B)** Distance in the central area in the OFT. **(C)** Time in the central area in the OFT. **(D)** Total distance in the EPM test. **(E)** Percentage of time in the open arm in the EPM test. **(F)** Percentage of open-arm entry times in the EPM test. **(G)** Composite aggressive score before the drug administration. **(H)** Composite aggressive score after the drug administration. PMDD-RIP, premenstrual dysphoric disorder-resident intruder paradigm; OFT, open-field test; EPM, elevated plus maze; Flx, fluoxetine; Pae, paeonol; *p < 0.05 compared with the control group; ***p < 0.001 compared with control group; ^#^p < 0.05 compared with the PMDD-RIP group; ^##^p < 0.01 compared with the PMDD-RIP group; ###p < 0.001 compared with the PMDD-RIP group; ^####^p < 0.0001 compared with the PMDD-RIP group. Twelve animals were used in each group.

To determine the effect of paeonol on PMDD-RIP rats, the OFT and EPM and composite aggressive tests were performed in each group after the final drug administration (on the 28^th^ day after ovariectomy). In the OFT (**Figures 9A–C**), paeonol did not show any significant effect, while fluoxetine reversed the central area time (p=0.0358) compared with PMDD-RIP rats. In the EPM test (**Figures 9D–F**), 12.11 mg/kg paeonol and fluoxetine both significantly increased the OT% (p=0.0007 and 0.0090) and OE% (p=0.0202 and 0.0425), indicating an obvious anti-anxiety effect. Moreover, the composite aggressive scores before (16^th^ day after ovariectomy) and after administration (28^th^ day after ovariectomy) are shown in **Figures 9G, H**. The results showed that drug treatment including fluoxetine (p=0.0020), 6.05 mg/kg paeonol (p < 0.0001), 12.11 mg/kg paeonol (p < 0.0001), and 24.23 mg/kg paeonol (p=0.0007) reversed the alterative composite aggressive score in PMDD-RIP rats.

## Discussion

Based on the above mentioned results, we conclude that in the dosage screening experiment, the optimal anti-anxiety dose of paeonol was identified as 17.5 mg/kg/d for 7 days. Paeonol could significantly improve the behavioural symptoms in PMDD rat models; however, the effective dose varied in different models, suggesting a dose-dependent effect. In PMDD-PWD model rats, 6.05 mg/kg paeonol treatment could significantly improve the anxiety and irritability, while treatment with 24.23 mg/kg paeonol showed anxiety-like effects in behavioural tests. In PMDD-RIP model rats, 12.11 mg/kg paeonol showed a superior effect in improving anxiety, particularly irritable emotional behaviour.

### 17.5 mg/kg Paeonol Is the Optimal Dosage Against Anxiety

Classical behavioural tests including the OFT, EPM, and LDB are simple and fast, and they are widely used in the initial screening of anti-anxiety drugs. Therefore, in this study, we selected these three methods to evaluate the drug effect and screen the optimal dosage of paeonol. The OFT is a method for evaluating the ability of autonomous movement and exploration of animals in a strange environment (25). On one hand, animals mainly move around the peripheral area with less activity in the central area because of the fear of the new environment. On the other hand, the inquisitive nature of animals leads them to attempt entry into the central area. Therefore, the anxiety state of animals could be evaluated (26). The EPM model is based on the spontaneous behaviour of animals, which has the advantages of simplicity and good reproducibility (27, 28). The conflicts of animals’ fear, exploration, and avoidance abilities were used to evaluate the anti-anxiety effects of the drugs. The LDB experiment was designed based on the fact that animals prefer darkness to light. Normally, rodents tend to stay in the dark, but their exploratory behaviour drives them to the light box. Therefore, the increase in the number and time of activities in the light box can reflect a reduction in animal anxiety (29, 30). Using the reliable behavioural evaluation system mentioned previously, we effectively screened 17.5 mg/kg as the optimal anti-anxiety effective intervention dose in mice treated with different doses of paeonol. Furthermore, in the OFT, we found that a 140 mg/kg paeonol treatment for 7 days showed significant anxiety-inducing effects, indicating that the anti-anxiety effect of paeonol was dose-dependent.

The anxiolytic-like effect of paeonol in mice has been confirmed previously in Xiao et al.’s study (31), wherein they found that the motor ability in animals were significantly limited with an increase in the dosage of diazepam. In our results, diazepam demonstrated an anxiety-causing effect in the OFT but an anxiolytic-like effect in the EPM and LDB tests. This conflicting phenomenon is possibly related to the drug dose. A previous report indicated that at a low dose, diazepam produced an anti-anxiety effect and improved the animals’ exploration ability in the central region, but at a high dose, it demonstrated a sedative effect (32). However, our research showed that a high dose of paeonol had no effect on the animals’ motor ability, which could be concluded from all kinds of behavioural tests. This indicates that the side effect of paeonol is less than that of diazepam, which provides a new option for clinical anti-anxiety applications.

### The Reasonability of Modelling Methods and Validity Analysis of Animal Models

Through clinical trials, Lotta Andréen et al. found that after long-term exposure to progesterone followed by withdrawal, the progesterone metabolite 3α-5α-tetrahydroprogesterone decreased γ-aminobutyric acid (GABA)-gated current by increasing the GABA_A_R α4 subunit, resulting in a change in the composition of the GABA_A_R subunit, thus reducing the inhibition of GABA in the central nervous system (33). Yan Li et al. confirmed that a sudden progesterone withdrawal could change the expression of GABA_A_R, increase the excitability of GABA in the central nervous system, and induce anxiety-like behaviour during a period of 24–72 h after stopping exogenous hormone injections (16). Because of the pathogenesis homology between the anxiety-like behaviour of the PMDD-PWD animal models and the anxiety symptoms in clinical PMDD patients, this study adopted the progesterone withdrawal paradigm to create a PMDD rat model for exploring the possible effects of paeonol. We found that rats showed a typical anxiety-like behaviour after the withdrawal of long-term progesterone injection, while fluoxetine, a first-line treatment for PMDD, alleviated the abnormal behavioural changes related to the oestrous cycle in model rats, which was consistent with the report of Machado Figueiredo et al. (8). Furthermore, in the 6.05 mg/kg dose group, paeonol could also significantly improve the anxiety and restlessness-like mood in PMDD-PWD model rats. All the evidence mentioned above show that a PMDD-PWD rat model has good surface validity and predictive validity.

RIP makes use of the territorial behaviour of resident rats in their home cages. When facing the invasion of exotic animals, resident animals face inexplicable pressure and show irritability and defensive behaviour. The psychology of this defensive behaviour in rodents is related to human anxiety, which simulates the irritable emotional expression in patients with clinical PMDD (34). In the aggressive behavioural test, the animals in the model group showed cycle-dependent changes in the composite aggressive score during the non-receptive period, while there were no significant changes in the composite aggressive score of animals in the control group before and after administration, indicating that the PMDD rat model was reliable and solid, which reproduced and verified the findings of Hoi-Por Ho et al. (18). Fluoxetine could significantly reduce the aggressive behaviour in animals, which proved that a first-line treatment of PMDD could improve the irritable mood of PMDD (21). In addition, each dose group of paeonol can significantly improve the irritable mood in PMDD animals after treatment, which enriches the performance of paeonol in the treatment of emotional diseases.

### Paeonol Could Improve the Behavioural Symptoms of PMDD Rat Models

In previous studies, paeonol was proven to play remarkable roles in numerous central nervous system disorders including diabetic encephalopathy, cerebral ischaemic injury, Alzheimer’s disease, Parkinson’s disease, ageing, and depression (35). The neuroprotective effect of paeonol may be related to the reduced release of neurotoxic and proinflammatory factors and the inhibitory effects of neuroinflammation (10, 11, 36, 37). However, in the treatment of PMS/PMDD, the pharmacodynamic evaluation of paeonol has not yet been reported. To address this issue, we established two classical and recognised PMDD models and observed the effect of paeonol on the behavioural symptoms.

As the anxiety-like emotional behaviour in the animal model of PWD is similar to the pathogenesis of anxiety in patients with clinical PMDD, in this study, PWD was used to establish the PMDD-PWD model (16). We found that 6.05 mg/kg paeonol could significantly improve the anxiety and irritability in PMDD-PWD model rats, while a dosage of 24.23 mg/kg demonstrated anxiety-like effects in the behavioural tests. Thus, the dose-dependent effect of paeonol was reconfirmed.

For the clinical diagnosis criteria of PMDD, a regular menstrual cycle is required with symptoms of irritability occurring over at least three menstrual cycles (1). However, in experimental research, animals with three consecutive oestrous cycles are rare, and the emotional cycle of animals is easily affected by the environment, diet, sleep, emotion, and other factors. In order to eliminate this challenge in the study, along with previous research (18), exogenous hormone supplementation was administered to induce the normal oestrous cycle after the ovaries were removed. Then, the PMDD-RIP model was established using a resident intruder to simulate the clinical performance of irritable emotional symptoms. Firstly, we found that the symptoms in the PMDD-RIP model rats were oestrous cycle-dependent. Irritability was obvious during the dioestrus I phase (non-receptive phase), which is consistent with the performance of clinical PMDD patients in the premenstrual period. Secondly, for these typical symptoms, such as the irritable mood symptoms of PMDD-RIP rats, 12.11 mg/kg paeonol showed an effective improvement.

Thus, with these two rat models, the anti-PMDD property of paeonol and its few side effects were confirmed in our study. As the first-line treatment in PMS/PMDD patients, the antidepressant, SSRI, is associated with obvious side effects. Thus, there is an urgent need to develop new drug treatment methods to improve the therapeutic measures for PMS/PMDD (5). Traditional herbal medicine has been used widely, and studies have shown that many traditional Chinese medicines or its components have anti-depressant or anti-anxiety effects (38–41). However, for PMS/PMDD treatment, reports on the effect of traditional Chinese medicine are rare. In addition to the anti-inflammatory, anti-oxidant, and neuroprotective properties of paeonol confirmed in previous studies (10, 35), the anti-PMDD effect of paeonol in this study may be concluded.

### The Effective Dose of Paeonol Varied in Different PMDD Models

Based on the abovementioned conclusions, paeonol could evidently improve the behavioural symptoms in both PMDD-PWD and PMDD-RIP rat models, but the effective dose varied. For PMDD-PWD rats, 6.05 mg/kg paeonol showed a significant treatment effect on PMDD-like symptoms, while for PMDD-RIP rats, 12.11 mg/kg was the optimal dosage. As the two PMDD models correspond to different pathogeneses, the mechanism by which paeonol works may vary. The underlying mechanism of this phenomenon remains to be further explored. Currently, the most popular hypothesis for interpreting the PMDD pathogenesis is associated with the GABA-A receptor; however, this is still being debated. Determining and confirming the pathophysiological alterations in PMS/PMDD is critical in this area, which can probably help in the interpretation of our findings. Moreover, paeonol has a variety of pharmacodynamic effects including anti-inflammatory, anti-tumour, neuroprotective, and anti-cardiovascular disease effects (35). The specific mechanism by which paeonol administration affects PMDD is still unclear, warranting further research for confirmation.

### The Effect of Paeonol Was Dose-Dependent

In the anti-anxiety effect experiment with mice or in the effect of paeonol on PMDD rats, it was easy to determine that a relatively high dose could cause anxiety-like behaviour, and conversely, an appropriate dose could relieve anxiety significantly. As a type of benzodiazepine, the potential target of paeonol are the benzodiazepine sites that bind to GABA-A receptors, and then, the Cl^−^ current of GABA-A receptor is mediated to increase the release of GABA (42). Different doses of paeonol may change the GABA-A receptor-gated current differently owing to the varied metabolism in the central nervous system. In this study, we only preliminarily confirmed the improvement effect of paeonol on the behavioural symptoms of PMDD, failing to provide a complete chain of evidence to interpret the underlying mechanism.

Other issues that were not investigated, which might affect the integrity of this study should be mentioned. First of all, this study failed to further explore the possible underlying mechanisms of drug intervention in model animals, which is necessary to understand and explain the occurrence of disease and drug effects. In fact, we also detected changes in hormones and neurotransmitters in the brain and peripheral blood, and failed to obtain valuable positive results. This finding is consistent with the finding that no valuable biomarkers were found in clinical patients and animal models with PMDD (43, 44). This is one of the puzzling problems in this field. The key to this problem may be the subtyping of PMDD (45–47). Different subtypes of PMDD may be mixed together, resulting in no valuable molecular clues. The animal models used in this article are typically symptom-mixed. This suggests that we should perform more in-depth and detailed studies on the heterogeneity identification of PMDD animal models in the future. Secondly, there is adequate evidence to suggest that the key mechanism of PMDD is the sensitivity of specific emotional regulation of the brain regions to the fluctuation of progesterone metabolites in different periods of the menstrual cycle (oestrous cycle) (7, 48), which has been revealed to some extent in previous studies (21), but not in-depth. In our follow-up work, we will use paeonol, an active monomer of traditional Chinese medicine that has been proved to be effective against PMDD, to interfere with model animals, and then observe the changes in brain sensitivity, to explore and confirm the possible central mechanism of paeonol.

## Conclusions

In this study, first, the anti-anxiety effect of paeonol was verified by using comprehensive behavioural tests, and the optimal dose was identified as 17.5 mg/kg/d for 7 days. Secondly, using two different PMDD rat models (PMDD-PWD and PMDD-RIP), the anti-PMDD effect of paeonol was concluded as follows: (i) paeonol could significantly improve the anxious and agitated emotion-like behaviour in PMDD rat models, but the effective dose varied in different models. (ii) The effect of paeonol on PMDD is dose-dependent; a high dose could cause anxiety-like behaviour, while an appropriate dose could relieve anxiety. Although additional investigation is still needed, our study indicates that paeonol is a potential therapeutic compound for PMS/PMDD and a new drug option that can help establish treatment dosage guidance.

## Data Availability Statement

PS, SW and MQ are responsible for presenting data supporting the results reported in the current study when required.

## Ethics Statement

The animal study was reviewed and approved by the Ethics Review Board of Shandong University of Traditional Chinese Medicine (No. DWSY201703013).

## Author Contributions

HZ and XG performed all experiments. ZL, YL, KX, HW and JX provided key assistance. PS, SW and MQ directed the project, performed statistics, interpreted data and finished the manuscript.

## Funding

The study was supported by the National Natural Science Foundation of China (No. 81974553) the Ministry of Science and Technology (No. 2017ZX09301064001), the Natural Science Foundation of Shandong Province (No. ZR2019MH053 and ZR2018BC024), and the Youth Innovation Team of Shandong Provincial Department of Education (No. 2019KJK002).

## Conflict of Interest

The authors declare that the research was conducted in the absence of any commercial or financial relationships that could be construed as a potential conflict of interest.

## Abbreviations

CE, closed arm entry times; CMC-Na, sodium carboxymethyl cellulose; CT, time in the closed arm; EPM, elevated plus maze; LDB, light dark box; OE, open-arm entry times; OFT, open-field test; OT, time in the open arm; PMDD, premenstrual dysphoric disorder; PMS, premenstrual syndrome; PWD, progesterone withdrawal; RIP, resident intruder paradigm; SSRIs, selective serotonin reuptake inhibitors.
